# Joint species distribution models of Everglades wading birds to inform restoration planning

**DOI:** 10.1371/journal.pone.0245973

**Published:** 2021-01-28

**Authors:** Laura E. D’Acunto, Leonard Pearlstine, Stephanie S. Romañach

**Affiliations:** 1 U.S. Geological Survey, Wetland and Aquatic Research Center, Fort Lauderdale, FL, United States of America; 2 Everglades National Park, National Park Service, Homestead, FL, United States of America; Universidad Austral de Chile, CHILE

## Abstract

Restoration of the Florida Everglades, a substantial wetland ecosystem within the United States, is one of the largest ongoing restoration projects in the world. Decision-makers and managers within the Everglades ecosystem rely on ecological models forecasting indicator wildlife response to changes in the management of water flows within the system. One such indicator of ecosystem health, the presence of wading bird communities on the landscape, is currently assessed using three species distribution models that assume perfect detection and report output on different scales that are challenging to compare against one another. We sought to use current advancements in species distribution modeling to improve models of Everglades wading bird distribution. Using a joint species distribution model that accounted for imperfect detection, we modeled the presence of nine species of wading bird simultaneously in response to annual hydrologic conditions and landscape characteristics within the Everglades system. Our resulting model improved upon the previous model in three key ways: 1) the model predicts probability of occupancy for the nine species on a scale of 0–1, making the output more intuitive and easily comparable for managers and decision-makers that must consider the responses of several species simultaneously; 2) through joint species modeling, we were able to consider rarer species within the modeling that otherwise are detected in too few numbers to fit as individual models; and 3) the model explicitly allows detection probability of species to be less than 1 which can reduce bias in the site occupancy estimates. These improvements are essential as Everglades restoration continues and managers require models that consider the impacts of water management on key indicator wildlife such as the wading bird community.

## Introduction

The Florida Everglades is a unique ecosystem of international importance in the United States. This large (28,000 km^2^) sub-tropical wetland supports many plant and wildlife species, provides freshwater to south Florida’s densely populated cities, and serves as protection against flooding and damage caused by high-intensity storms such as hurricanes [1]. Once a vast wetland that extended across all south Florida, the hydrology of the Everglades has been altered dramatically to meet human needs [2]. In 2000, the Comprehensive Everglades Restoration Plan (CERP) was authorized by Congress in order to restore this ecosystem to a hydrologic regime more reflective of its historic flow while meeting the water supply and flood protection needs of south Florida [3]. CERP is one of the largest ongoing restoration projects in the world and requires continuing science that seeks to understand the ecosystem and its function so that restoration efforts are successful.

Those involved in making Everglades restoration decisions rely on ecological models that forecast the expected responses of key indicator species to proposed projects or water management operations. One such indicator is the presence of wading bird (egret, ibis, stork, heron, and spoonbill) colonies across the system [4]. In the pre-drainage Everglades system, tens of thousands of nesting wading birds were common across the landscape during their breeding season, but populations declined precipitously after draining of the system began [5, 6]. The decline of wading birds served as an important motivation to begin restoration of the Everglades and their return to historic numbers and colony locations is considered a sign of successful restoration [4]. Thus, models that can predict wading bird response to anticipated changes in hydrologic patterns from restoration projects are highly valuable decision support tools for restoration decision makers.

Currently, wading bird response to water management in the Everglades is predicted using a suite of Wading Bird Distribution Evaluation Models (WADEM) for the great egret (*Ardea alba*), wood stork (*Mycteria americana*), and white ibis (*Eudocimus albus*) [7]. WADEM is a species distribution model that uses relationships derived by regressing the number of birds in a spatial cell to the average hydrologic conditions in that cell over a decade-long time scale. Because each species was modeled separately, the predicted suitability indices are on different scales, which can make it difficult for decision-makers to compare responses across species. Additionally, these models have not accounted for the probability of species detection and instead assume detection probability is 1 and that when species are present, they are detected. The literature has demonstrated that detection probability of a species can influence wildlife distribution estimates in significant ways and should be explicitly included in models when possible [8–12]. WADEM considers three species of interest within the Everglades, but nesting wading birds can include additional species such as roseate spoonbills (*Platalea ajaja*), glossy ibis (*Plegadis falcinellus*), snowy egrets (*Egretta thula*), tricolored herons (*Egretta tricolor*), little blue herons (*Egretta caerulea*), and great blue herons (*Ardea herodias*). Considering additional species that are both rare and more common across the landscape can provide a broader picture of the wading bird community response to altered hydrology in the Everglades [13]. Further, the presence of one species of wading bird could influence the presence of another species (via attraction or avoidance). These potential species interactions could be important drivers of wading bird distribution but have yet to be incorporated into Everglades wading bird species distribution models [14].

One way to address some of these shortcomings of WADEM is to model wading birds as a joint species distribution model (JSDM). JSDMs refer to a suite of models that vary in specific approach but accomplish a general goal: to model the distributions of species jointly in order to make inferences about a community. JSDMs can be generalized as coming from two perspectives: 1) multispecies occupancy models that account for imperfect species detection [15, 16] and 2) stacked single-species distribution models that account for residual correlation of species apparent occupancy due to biotic interactions or missing environmental covariates [17, 18]. In the former perspective, residual correlations of species occupancy are not explicitly modeled, while in the latter perspective, detection of species is assumed to be one. Tobler et al. [19] also developed a model that combined these two perspectives.

Considering these developments in the species distribution modeling literature, we sought to update the current model of Everglades wading bird distribution by building a joint species distribution model that accounts for species detection probability using a multispecies occupancy modeling framework. We had several goals in applying this approach: 1) to consider a larger suite of wading bird species when assessing proposed restoration projects; 2) to generate output for each species on the same scale of response for ease of comparison among species; and 3) to address imperfect detection within our model.

## Materials and methods

### Data

**Fig 1:**
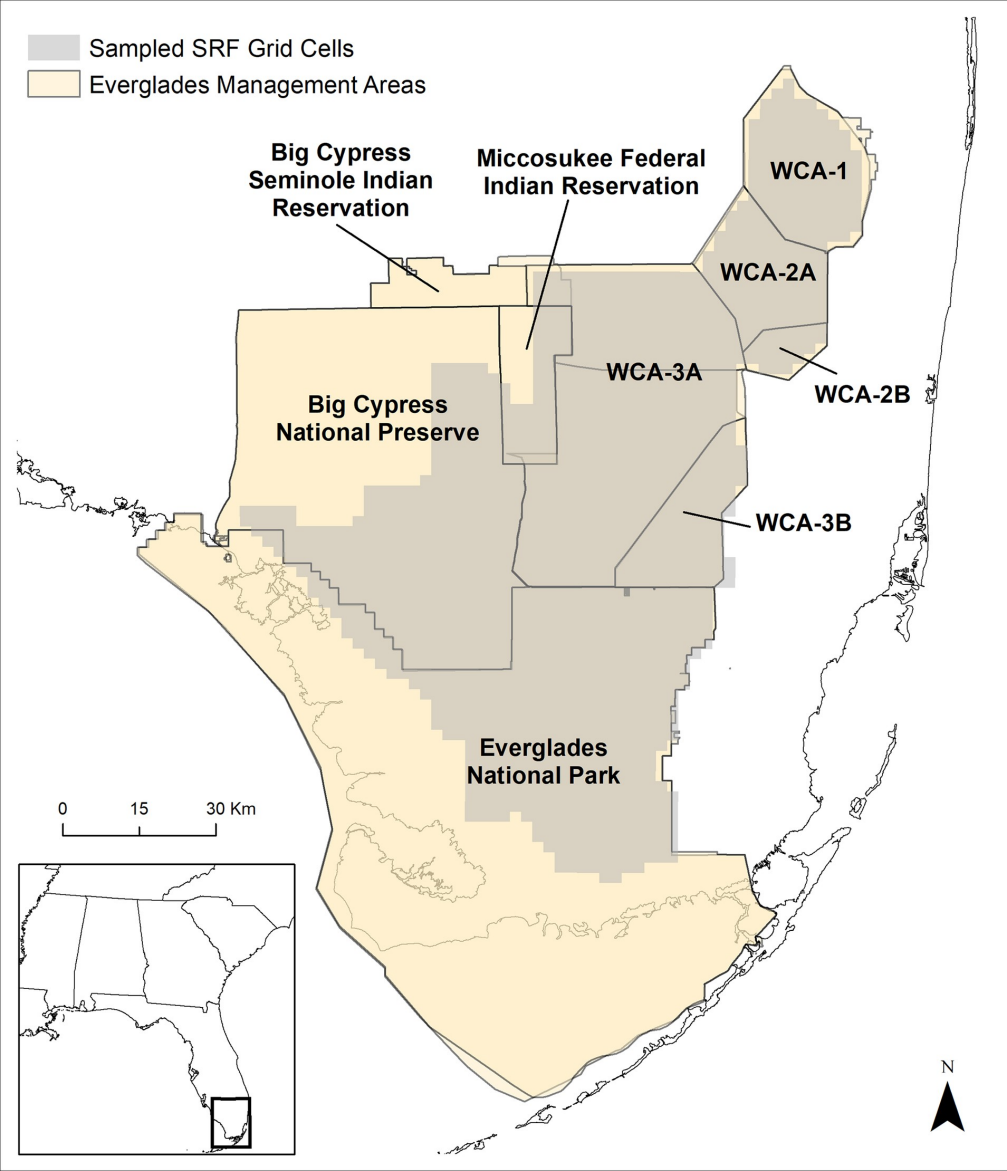
Sampling locations. Spatial extent of the Systematic Reconnaissance Flight (SRF) grid cells used to fit a joint species distribution model for wading birds and their location relative to protected areas within the southern Everglades. These areas include the Big Cypress Seminole Indian Reservation, the Miccosukee Federal Indian Reservation, Water Conservation Areas (WCA-1, 2A, 2B, 3A, and 3B), Big Cypress National Preserve (BCNP), and Everglades National Park (ENP). The grid shown is only those cells used in the model; the full extent of the SRF surveys is larger. Sources: Shapefiles for U.S. state boundaries available from census.gov. Shapefiles for the Everglades Management Areas provided by the Florida Fish and Wildlife Conservation Commission (https://gis.myfwc.com/Data/KMZ_files/Management%20-%20Wildlife%20Mgt%20Areas%20-%20Generalized%20-%20FL.kmz), the U.S. Fish and Wildlife Service (https://ecos.fws.gov/ServCat/Reference/Profile/128178) and the National Park Service (https://public-nps.opendata.arcgis.com/datasets/nps-boundary-1). Indian Reservation shapefiles provided by the U.S. Bureau of Indian Affairs (https://www.sciencebase.gov/catalog/item/4f4e4a2ee4b07f02db61576c).

Species detection-nondetection (species was observed or not observed) data were generated from Systemic Reconnaissance Flights (SRF) conducted between 2000–2009 across several regions of the greater Everglades: the Big Cypress Seminole and Miccosukee Federal Indian Reservations, the water conservation areas (WCAs), the southern portions of Big Cypress National Preserve (BCNP), and the north-central portions of Everglades National Park (ENP, Fig 1). These aerial surveys were conducted monthly between December and June of each year, corresponding to the general breeding season of wading bird species present in the system. The surveys are conducted on transects moving east-west along a 2-by-2 km grid. Flying at an altitude of ~61 m, two observers record the species and numbers of wading birds present within the grid cell. For more details on the methodology and history of the SRF transects, see Conroy et al. [20]. For grid cells that overlapped the extent of available hydrologic covariates, we developed detection histories for nine species of wading bird over the ten-year period: great blue heron, glossy ibis, great egret, little blue heron, roseate spoonbill, small dark herons (a species group mainly comprised of tricolored herons), small white herons (a species group mainly comprised of snowy egrets), white ibis, and wood stork. We assessed the presence or absence of each species in each SRF grid cell for the months of January, February, March, April, and May, creating a detection history of 5 visits where 0 indicated the species was not detected and 1 indicated the species was detected at least once within the grid cell. The number of grid cells we were able to generate detection histories for totaled 1,782 and were stacked by year, totaling 17,820 sampled locations included within the model.

### Covariates

Wading bird presence in the greater Everglades is driven by the availability of prey fishes and invertebrates [21]. During the wetter summer months, water within the landscape is deeper and the availability of prey to wading birds is restricted to smaller areas of the landscape. However, during the drier seasons which coincide with the breeding season, water typically slowly recedes and creates shallow pools where fish density is high, and birds can forage easily [22]. Thus, hydrologic conditions across the landscape can serve as a proxy for prey availability to wading birds and can predict wading bird presence. These responses can also be species-specific due to differences in species foraging strategy. For example, ibises, storks, and spoonbills are tactile feeders, using their beaks to ‘feel’ prey within the water. Conversely, egrets and herons are visual feeders, watching the water and striking prey [21, 23]. Ibises and storks therefore require higher densities of fish to successfully forage, which typically coincides with relatively low water depths compared to egrets and herons [24].

We selected six site covariates to include in the model that could explain wading bird occupancy (Table 1). Each hydrologic metric was calculated from daily water depth estimates generated by the Everglades Depth Estimation Network (EDEN) [25], which uses a network of water gages across the system to interpolate water depths at a 400 m resolution. Because our sample grid had a resolution of 2 km, we averaged the 400 m cells that fell into each 2 km cell as the final metric estimate. Depth during the breeding season described the foraging conditions present in that cell [26, 27]. Water recession rate served as an indicator of prey density, where a steady recession rate with minimal water reversals would produce dense prey throughout the breeding season to remain available to birds while nesting [28]. Finally, the number of days where surface water is absent over the past 3 years serves as an indicator of long-term hydrologic conditions within the grid cell which can impact both the vegetation community and crayfish populations within the system [29–31]. The quadratic effect of each hydrologic covariate was also included within the model. In addition to the three hydrologic variables, we included the landscape characteristic of proportion tree canopy cover as a habitat covariate [32]. Proportion tree canopy cover generated by the U.S. Forest Service was included as a proxy for vegetation structure across the landscape and an indicator of the availability of nesting substrate [33]. Finally, to address additional spatial processes in wading bird distributions not captured by hydrology or vegetation structure, we included the easting and northing coordinates of each grid cell as additional covariates of wading bird site occupancy. While temporal trends likely exist, we did not include inter-annual effects on probability of occupancy because we were interested in fitting generalized relationships between landscape covariates and probability of occupancy for each species that could be used to generate near- and long-term predictions of occupancy from future hydrologic scenarios.

**Table 1:** Site covariates used for predicting species occupancy.

Variable	Mean	SD	Range	Description
Breeding season depth	17.28	17.38	0.00–98.65	Average depth (cm) in the EDEN cell from January 1—June 30
Recession	0.02	0.13	-0.45–0.55	Water recession rate within the EDEN cell between January 1—June 30
Days Dry	235.20	262.26	0–1097	Number of days surface water is ≤ 0 cm in the EDEN cell from June 30 of the current year to three years prior.
Canopy cover	0.07	0.11	0.0–0.64	Proportion of tree canopy cover within the EDEN cell.
Easting	528048.8	22477.46	467021–575021	X coordinate of the centroid of the SRF grid cell
Northing	2863767	35566.75	2792200–2948200	Y coordinate of the centroid of the SRF grid cell

Site-specific covariates used for predicting wading bird species occupancy within a multispecies occupancy model. We report mean, standard deviation, and range of the values within the dataset analyzed.

### Modeling

We modeled Everglades wading bird distributions in response to hydrologic conditions using a joint species distribution model that accounted for imperfect detection. The model’s base is a hierarchical multispecies occupancy model [15, 34, 35] consisting of an ecological process model that describes the probability of occurrence at each site and the species detection process [8]. Using a Bayesian framework, we modeled all nine species simultaneously by drawing species-specific occupancy and detection relationships from a common prior distribution. The probability of occurrence is defined as *z_ji_~Bernoulli*(*ψ_ji_*) where *ψ*_*ji*_ is the probability that the site (an SRF grid cell) *j* is occupied by species *i* and *z*_*ji*_ is the occupancy state (0 for unoccupied and 1 for occupied) for site *j* and species *i*. Occupancy is thus modeled as a function of site-level covariates: *logit*(ψ_*ji*_) = *α*_ψ,i_+β_cov,i_*cov_j_… where *α*_*ψ*,*i*_ are the species-specific model intercepts, *β*_*cov*,*i*_ are the species-specific beta estimate explaining the relationship between species occupancy and the covariate of interest, and *cov*_*j*_ is the measurement of the covariate of interest at site *j*. The probability of detection was defined as *X_jki_~Bernoulli*(p_jki_*z_ji_) where *p*_*jki*_ is whether the species *i* is detected at site *j* during site visit *k* (1 for detected, 0 for not detected). We then modeled detection probability as *logit*(*p_jki_*) = *α_pi_*, where *α*_*pi*_ is the species-specific model intercept for detection probability.

Occupancy covariates (depth during the breeding season, recession rate, number of dry days, the quadratic effect of these three variables, canopy cover, easting, and northing) were modeled as species-specific random effects drawn from a community-level normally distributed prior (mean of 0 and precision of 0.001). Detection probability varied only among species, but not over time or by site-level covariates.

We ran the model in program R [36] and JAGS [37] via the R2jags package [38] with 20,000 iterations of 3 chains, a burn-in of 10,000, and a thinning rate of 10. We determined model convergence was achieved by visually examining the MCMC chains to ensure adequate mixing across iterations and by ensuring that the R-hat statistic measured < 1.1 for all parameters within the model [39]. Covariate coefficient estimates with 95% credible intervals that did not cross 0 were considered important predictors of species occupancy as this was reflective of a consistent relationship within model iterations. To assess the strength of the covariates on predicting occupancy of each species, we calculated Bayes factors [40] on each parameter using the Savage-Dickey ratio test [41] calculated using the R package ‘bayestestR’ [42]. Particularly strong Bayes factors can result in numbers several magnitudes larger than weaker ones, therefore we compared the natural log of the raw Bayes factors to facilitate comparison among species and covariates. Thus, natural log-transformed Bayes factors > 4.61 indicate decisive evidence for the importance of a covariate, while Bayes factors < 0.0 indicate a covariate that likely has very little impact on species occupancy [40]. Code used to fit the model is provided in the (S1 Appendix).

### Model validation and fit

We assessed model goodness-of-fit by calculating the Dunn-Smyth residuals and examined the values plotted against the species-specific fitted occupancy values [43, 44]. Typically, plots where the 95% confidence interval of the line of best fit from these residual plots crosses 0 indicates a model with good fit [44]. To assess model accuracy, we calculated species-specific area under the receiver operating characteristic curve (AUC) values. AUC ranges from 0 to 1, with values > 0.7 indicative of an adequately accurate model [45, 46].

### Predicted occupancy maps

We used the mean of the posterior for each parameter and the values of the landscape-scale variables to generate species-specific predicted probability of occupancy maps for the year 2009.

## Results

**Fig 2:**
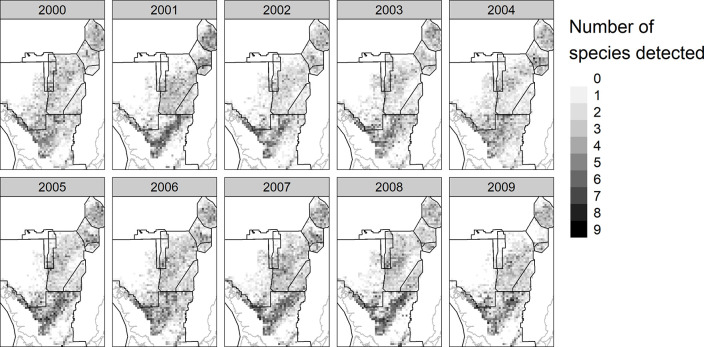
Species detections. Total number of species (out of a possible 9) detected in each SRF grid cell in each year across the model’s spatial extent.

The species used in the model ranged from relatively common (great egret and white ibis) to relatively rare (roseate spoonbill and glossy ibis). The species with the least number of detections was the roseate spoonbill with 381, while the highest number of detections was the great egret with 13,042 detections. The spatial distribution of each species also varied by year, with some years showing higher diversity within the sampled grid cells than in other years (Fig 2).

**Fig 3:**
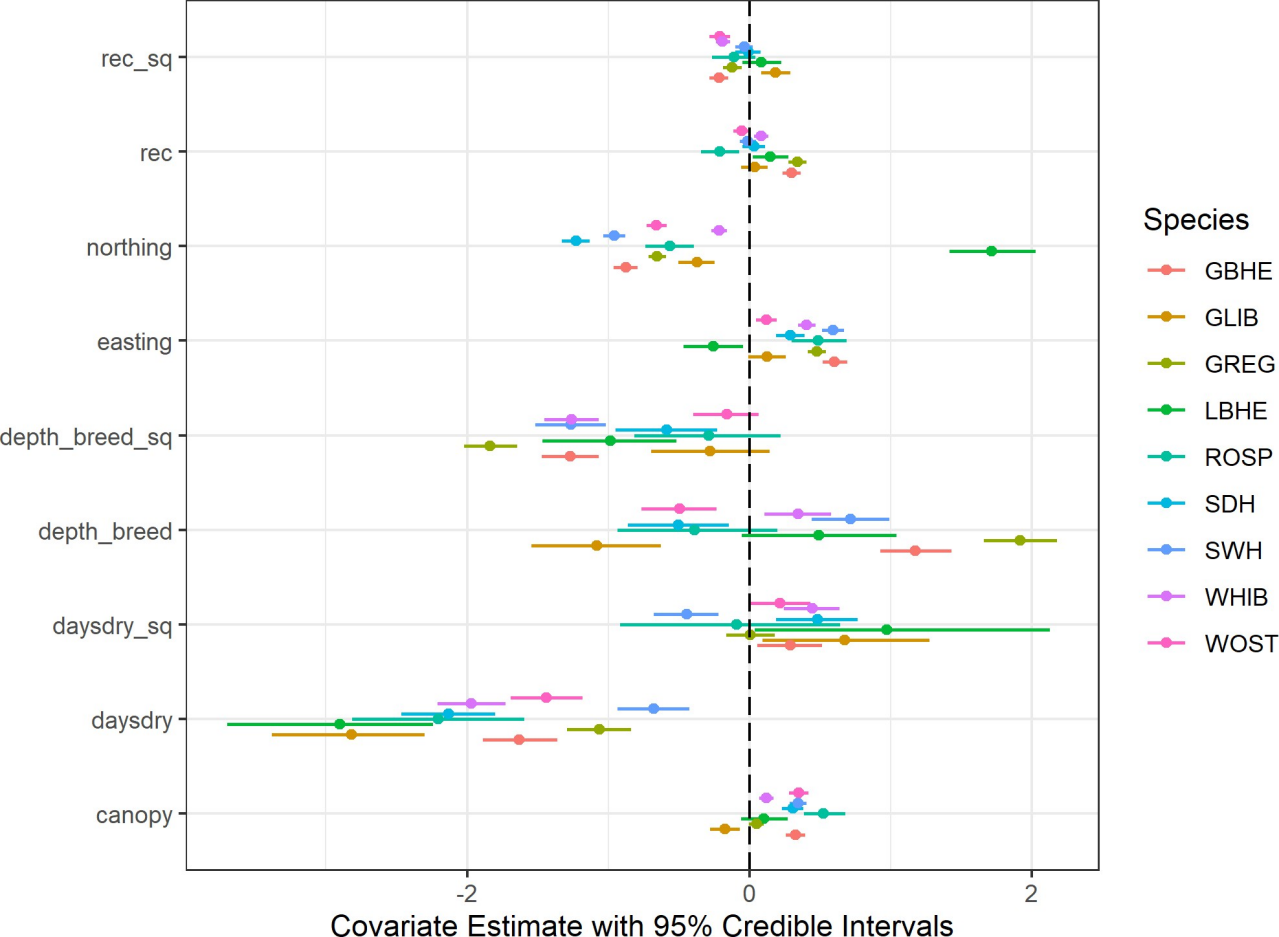
Covariate coefficient estimates. Covariate estimates for the site covariates (mean of the posterior and 95% posterior interval) based on SRF surveys conducted between 2000–2009 for 9 species resulting from a joint species distribution model accounting for imperfect detection. Species are: GBHE (great blue heron); GLIB (glossy ibis); GREG (great egret); LBHE (little blue heron); ROSP (roseate spoonbill); SDH (small dark herons); SWH (small white herons); WHIB (white ibis); and WOST (wood stork).

**Fig 4:**
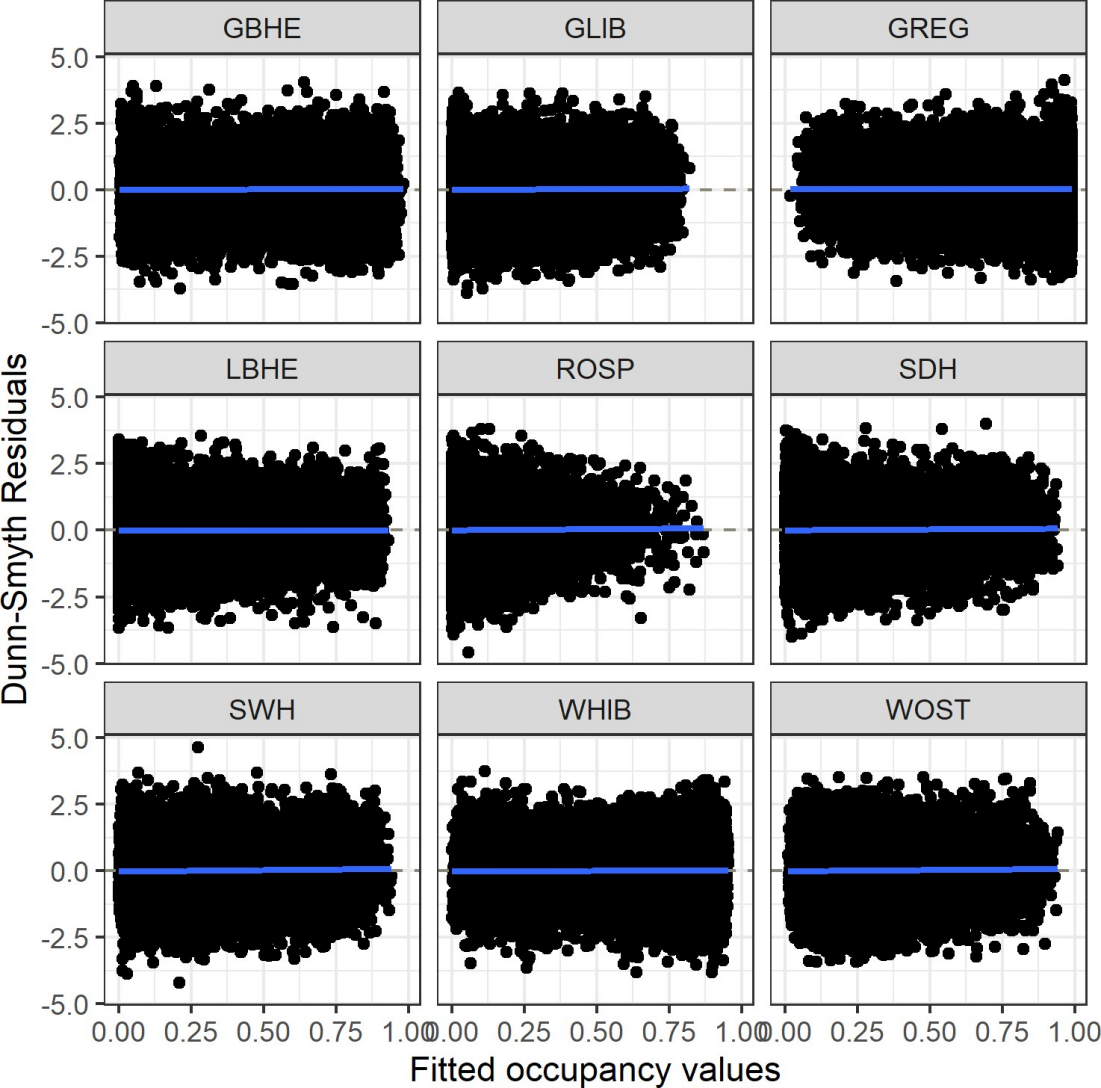
Dunn-Smyth residual plots. Dunn-Smyth residuals plotted against occupancy values fitted from a joint species distribution model of wading birds surveyed using Systematic Reconnaissance Flights from 2000–2009. If the fitted line and its 95% confidence interval overlaps 0, it is indicative of a well-fit model for that species. Species are: GBHE (great blue heron); GLIB (glossy ibis); GREG (great egret); LBHE (little blue heron); ROSP (roseate spoonbill); SDH (small dark herons); SWH (small white herons); WHIB (white ibis); and WOST (wood stork).

The joint species distribution model provided species-specific relationships between site covariates and occupancy (Fig 3) along with species-specific detection probability (Table 2). The average R-hat statistic for all parameters in the model was 1.01, less than the threshold of 1.1 above which a model is likely not fully converged. Additionally, visual examination of the MCMC draws did not show evidence of a lack of convergence within the model. The Dunn-Smyth residual plots for each species indicated adequate fits for all species (Fig 4) as evidenced by no strong trend in the residuals plotted against fitted occupancy values. Species-specific mean AUC values ranged from 0.712 to 0.848, with all species achieving AUC values > 0.70 indicating adequate model accuracy (Table 2).

**Table 2:** Species-specific detection probabilities and AUC.

	Detection probability			AUC		
Species	Mean	Low	High	Mean	Low	High
Great blue heron	0.179	0.173	0.184	0.755	0.754	0.756
Glossy ibis	0.059	0.051	0.067	0.750	0.748	0.751
Great egret	0.511	0.506	0.514	0.848	0.847	0.849
Little blue heron	0.042	0.034	0.051	0.837	0.835	0.838
Roseate spoonbill	0.042	0.032	0.054	0.771	0.765	0.775
Small dark herons	0.131	0.123	0.139	0.794	0.793	0.795
Small white herons	0.168	0.161	0.175	0.732	0.731	0.734
White ibis	0.356	0.351	0.361	0.785	0.784	0.786
Wood stork	0.151	0.143	0.157	0.712	0.710	0.713

Species-specific wading bird detection probabilities and AUC (mean of the posterior distributions and 95% posterior intervals) estimated by a joint species distribution model fit using data from Systematic Reconnaissance Flight surveys conducted between 2000–2009.

**Fig 5:**
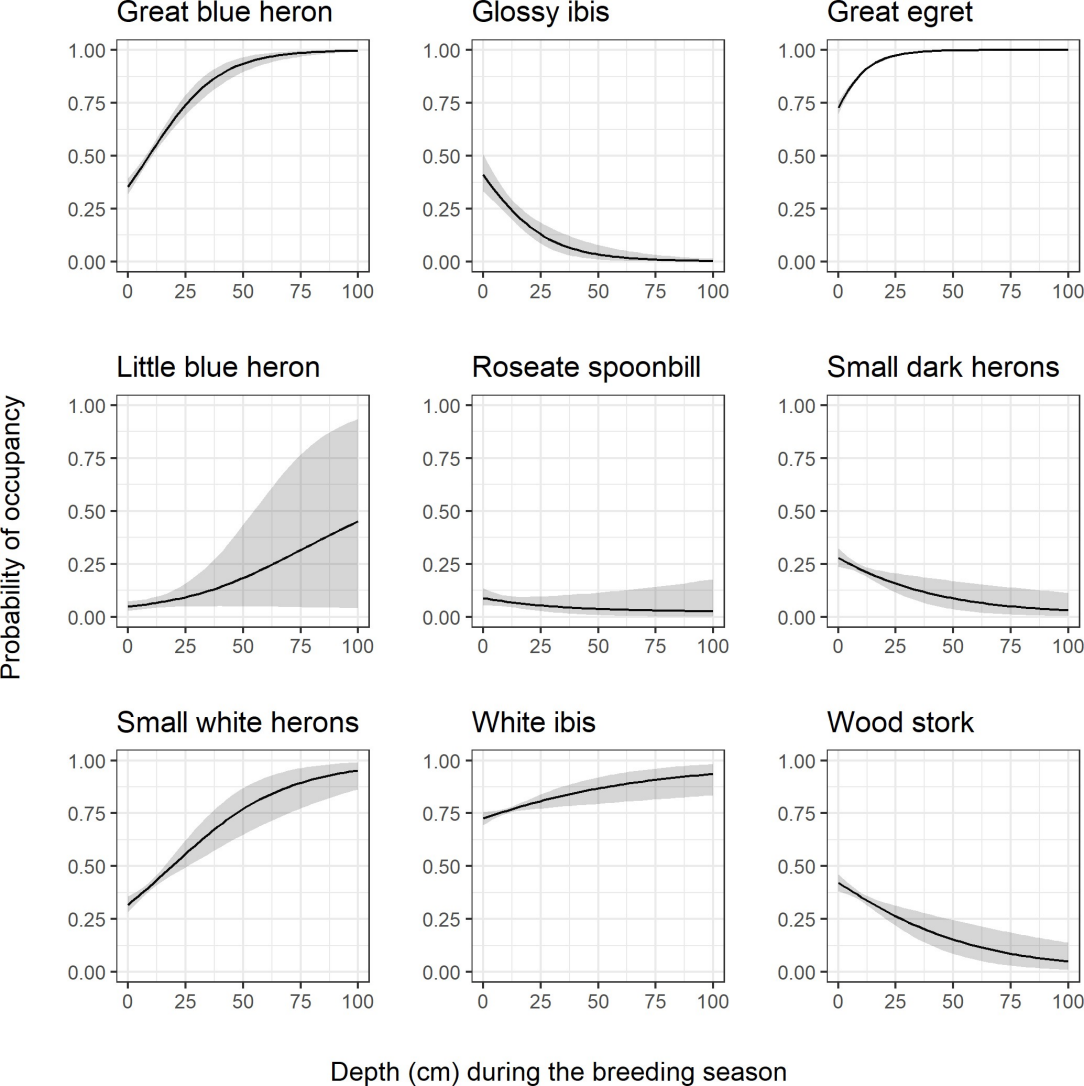
Breeding season depth response curves. Species-specific relationships between probability of occupancy and average depth during the breeding season (Jan 1 –June 30) from a fitted joint species distribution model of wading birds. Responses are calculated with all other model variables held at their mean. The black line represents the posterior mean and the gray shading represents the 95% posterior interval.

Breeding season depth was a particularly strong predictor of occupancy for the great blue heron, glossy ibis, great egret, and roseate spoonbill based on calculated parameter-specific Bayes factors (Table 3, values > 4.61). We generated species-specific response curves for each habitat covariate where the 95% credible interval did not cross 0. When all other variables are held at their mean, probability of occupancy varied across species with the average water depth during the breeding season (Fig 5). Great blue herons, little blue heron, great egrets, small white herons, and to a lesser extent, white ibis all showed a greater probability of occupancy at water depths > 25 cm, while the glossy ibis, small dark herons, wood stork and to a lesser extent the roseate spoonbill showed a lower probability of occupancy at water depths > 25 cm.

**Table 3:** Species-specific Bayes factors for parameters predicting species occupancy.

Parameter	Species								
	GBHE	GLIB	GREG	LBHE	SDH	SWH	ROSP	WHIB	WOST
Depth	*16*.*25*	*8*.*37*	*38*.*44*	-0.72	-1.34	1.16	*5*.*19*	0.57	2.54
(Depth)^2^	*21*.*03*	-1.83	*40*.*67*	*4*.*71*	-1.86	2.19	*17*.*99*	*25*.*34*	-2.36
Recession	*13*.*72*	-3.77	*23*.*30*	-1.11	0.88	-3.82	-4.51	0.22	-3.22
(Recession)^2^	*7*.*82*	2.46	0.73	-3.04	-2.66	-4.20	-3.77	*8*.*22*	*6*.*38*
Days Dry	*24*.*49*	*24*.*88*	*15*.*74*	*21*.*07*	*13*.*13*	*33*.*77*	*6*.*91*	*34*.*03*	*17*.*55*
(Days Dry)^2^	-0.20	0.28	-3.58	0.21	-2.09	1.92	2.82	*4*.*82*	-1.18
Canopy	*18*.*42*	0.61	-2.78	-2.90	*12*.*38*	*9*.*64*	*20*.*62*	3.44	*20*.*58*
Easting	*26*.*58*	-2.08	*32*.*13*	-0.49	*8*.*72*	*6*.*14*	*36*.*42*	*23*.*22*	0.91
Northing	*46*.*06*	*13*.*53*	*32*.*55*	*27*.*69*	*9*.*20*	*53*.*61*	*40*.*73*	*9*.*47*	*36*.*72*

Natural log-transformed Bayes factors for each species-specific parameter influencing species probability of occupancy using the posterior distributions from a fitted joint species distribution model of Everglades wading birds. Italicized numbers are those Bayes factors > 4.61 which indicate decisive evidence of that parameter influencing species occupancy probability. Species are: GBHE (great blue heron); GLIB (glossy ibis); GREG (great egret); LBHE (little blue heron); ROSP (roseate spoonbill); SDH (small dark herons); SWH (small white herons); WHIB (white ibis); and WOST (wood stork).

**Fig 6:**
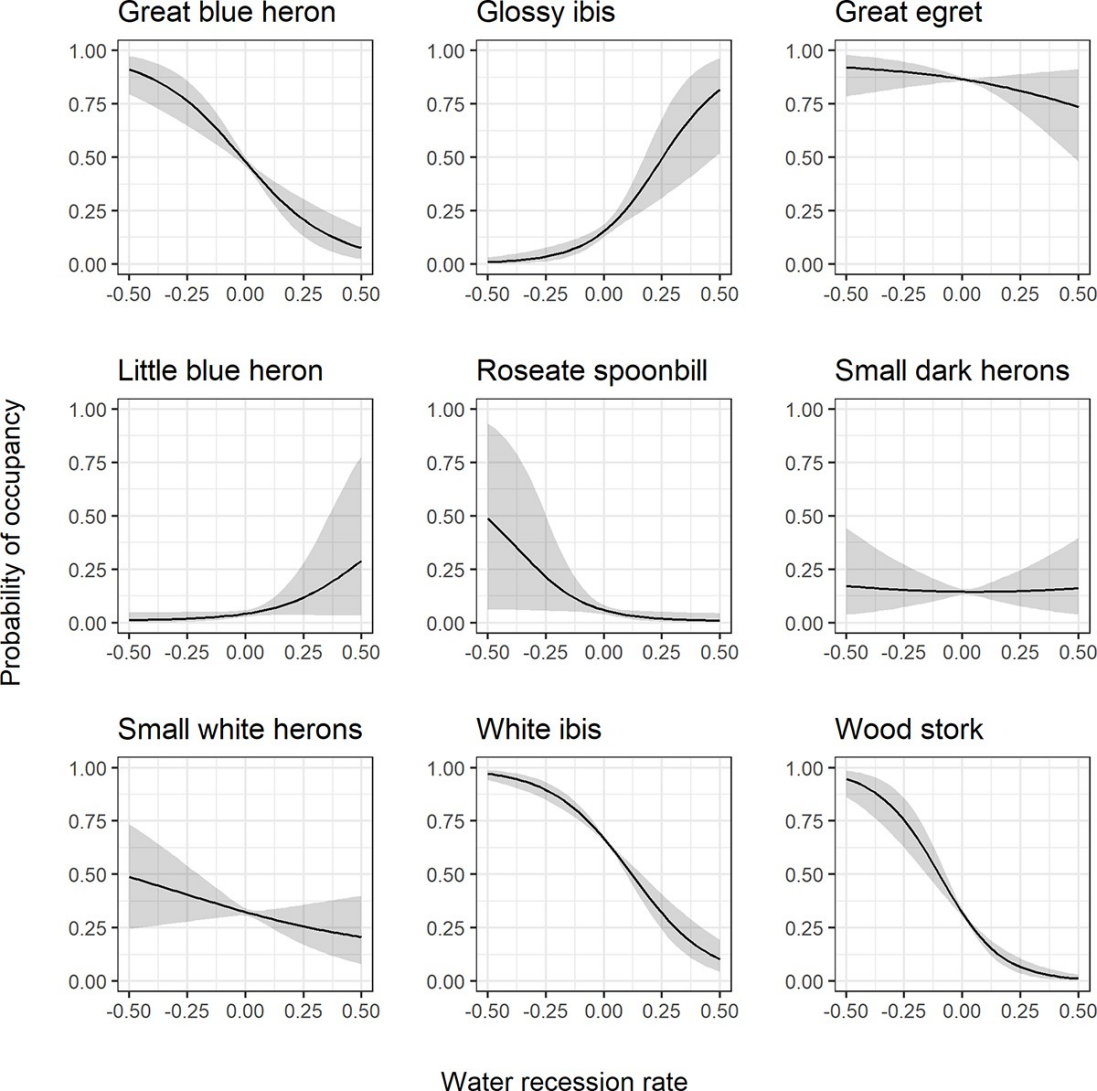
Water recession rate response curves. Species-specific relationships between probability of occupancy and average water recession rate during the breeding season (Jan 1 –June 30) from a fitted joint species distribution model of wading birds. Responses are calculated with all other model variables held at their mean. The black line represents the posterior mean and the gray shading represents the 95% posterior interval.

Probability of occupancy as a function of water recession rate during the breeding season also varied by species (Fig 6). The calculated Bayes factors for the water recession parameters indicated that water recession rate’s influence on probability of occupancy was strongest for the great blue heron, great egret, white ibis, and wood stork (Table 3, values > 4.61). The great blue heron, great egret, white ibis, roseate spoonbill, and wood stork had the greatest probability of occupancy when water recedes an average of 0.5 cm per day, with occupancy decreasing as the recession rate moves toward values indicative of water depth increases and not recession per se. However, glossy ibis and little blue herons exhibited the opposite trend, where probability of occupancy decreased as water approached receding and average of around 0.5 cm per day. Small white herons, great egret, and small dark herons did not show a particularly strong relationship with water recession rate, though the relationship was consistent within the model.

**Fig 7:**
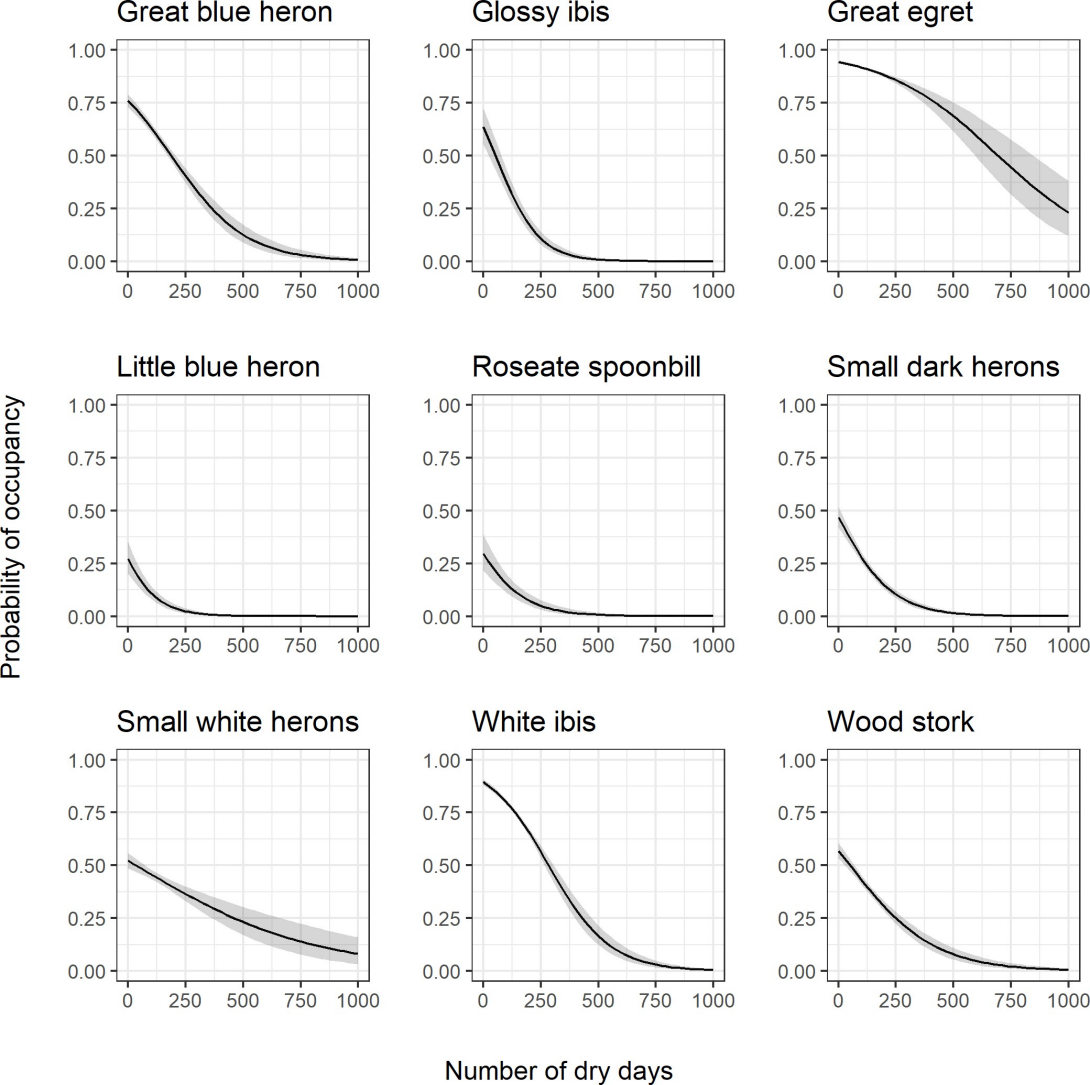
Number of dry days response curves. Species-specific relationships between probability of occupancy and number of dry days over the last 3 years from a fitted joint species distribution model of wading birds. Responses are calculated with all other model variables held at their mean. The black line represents the posterior mean and the gray shading represents the 95% posterior interval.

Probability of occupancy as a function of the number of dry days over the previous 3 years followed a similar trend across all modeled species (Fig 7) and calculated Bayes factors indicated that this variable was a strong predictor of occupancy for all species (Table 3, values > 4.61). While the maximum probability of occupancy varied, each species was more likely to occur in grid cells where the number of dry days was < 300, with the peak always occurring at 0 dry days over the last three years.

**Fig 8:**
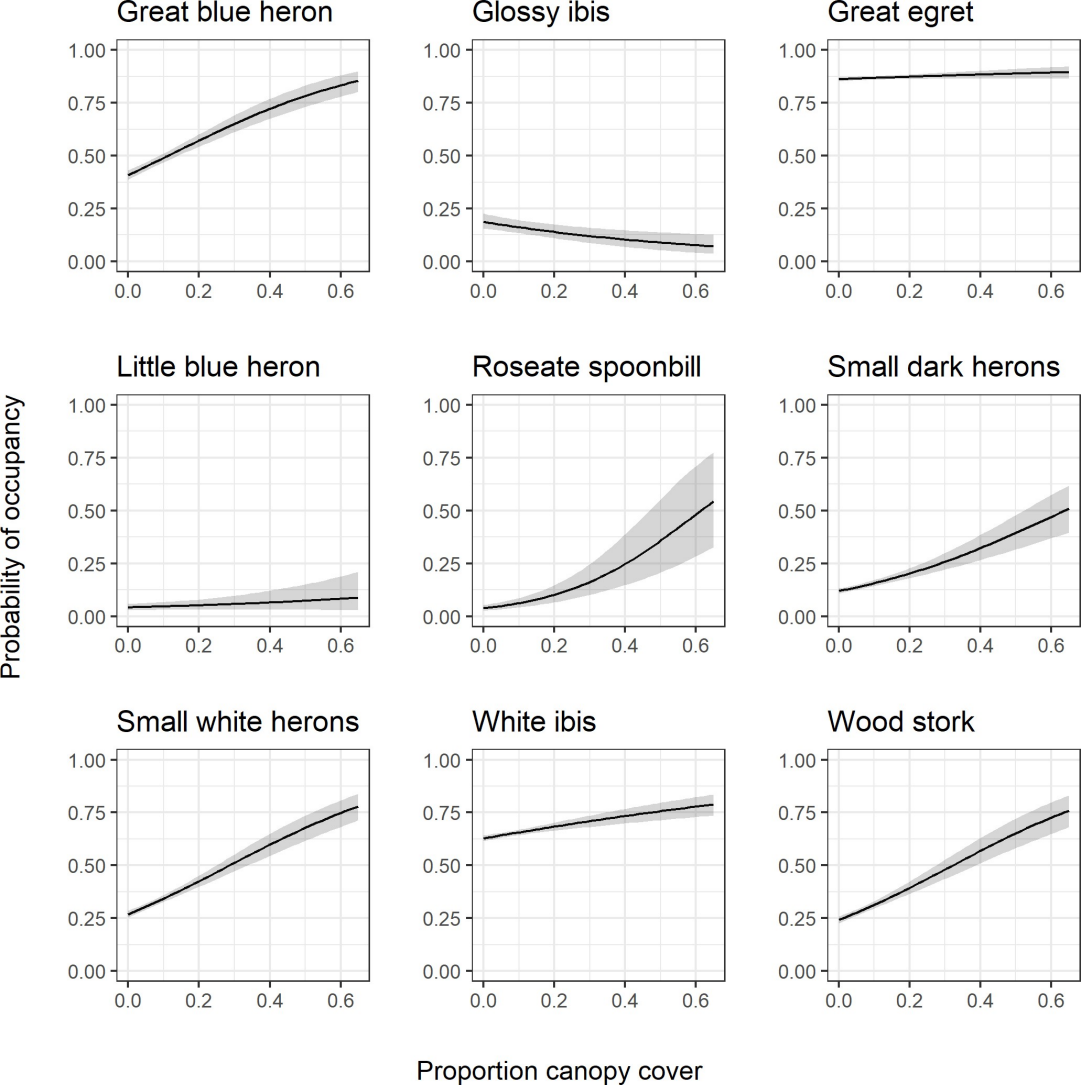
Canopy cover response curves. Species-specific relationships between probability of occupancy and proportion tree canopy cover fitted joint species distribution model of wading birds. Responses are calculated with all other model variables held at their mean. The black line represents the posterior mean and the gray shading represents the 95% posterior interval.

The calculated Bayes factors indicated that proportion of tree canopy cover was a particularly strong predictor of occupancy for the great blue heron, small dark herons, small white herons, roseate spoonbills, and wood stork (Table 3, values > 4.61). Probability of occupancy as a function of proportion tree canopy cover was consistent for all modeled species except the glossy ibis (Fig 8). For most species, probability of occupancy increased as canopy cover increased, but the magnitude of this effect varied across species. The glossy ibis showed an opposite response where probability of occupancy decreased as canopy cover increased.

**Fig 9:**
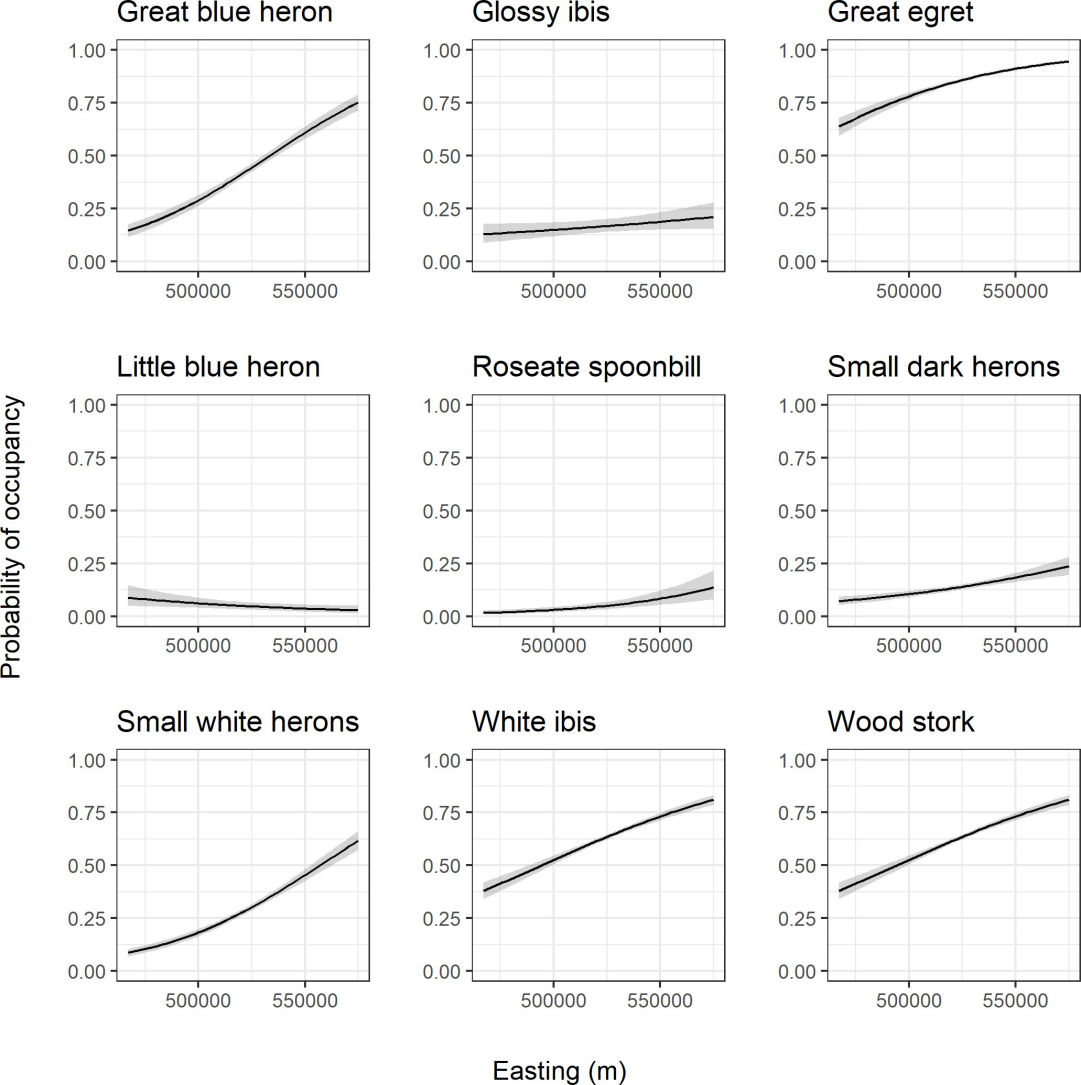
Easting coordinate response curves. Species-specific relationships between probability of occupancy and the easting coordinate from a fitted joint species distribution model of wading birds. Responses are calculated with all other model variables held at their mean. The black line represents the posterior mean and the gray shading represents the 95% posterior interval.

**Fig 10:**
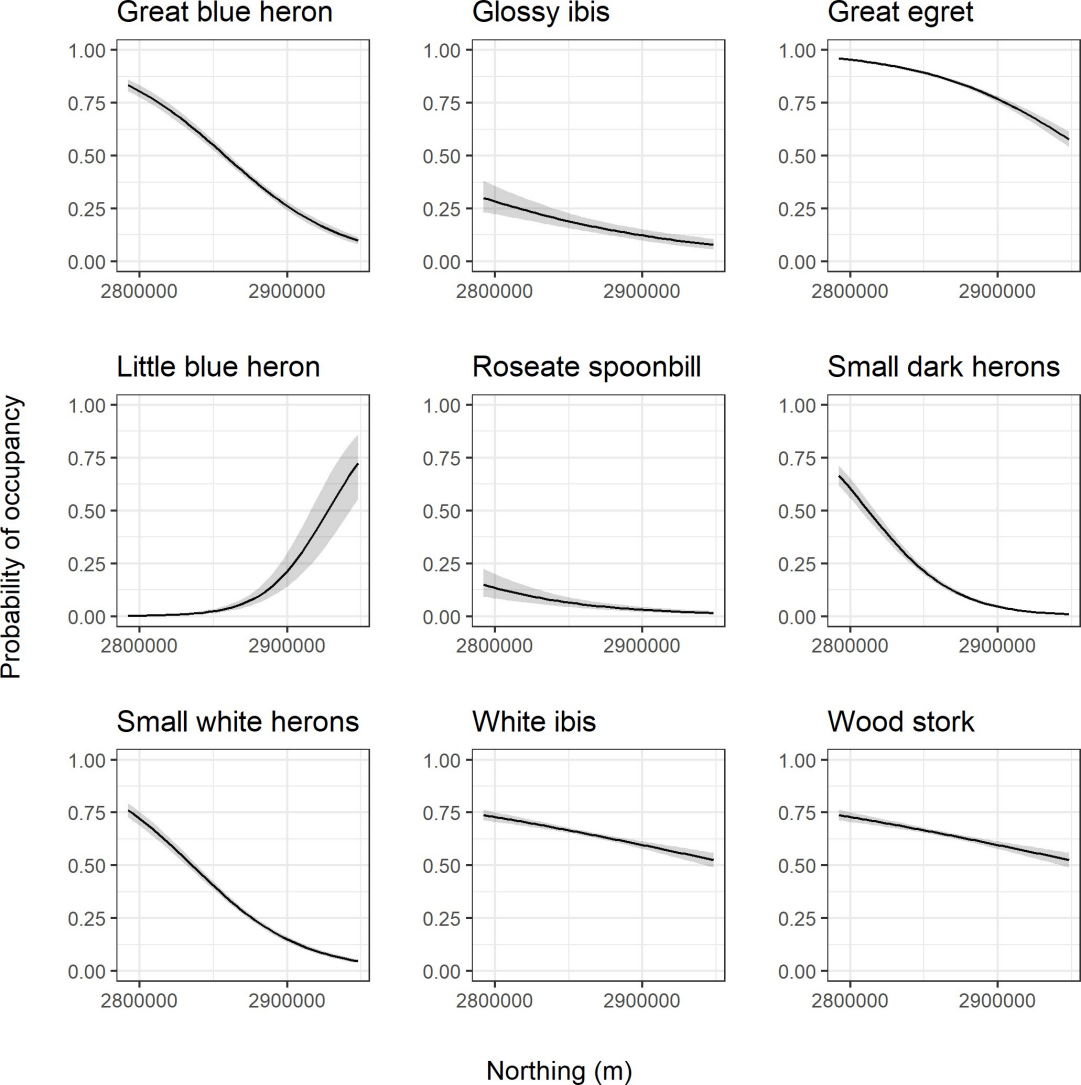
Northing coordinate response curves. Species-specific relationships between probability of occupancy and the northing coordinate from a fitted joint species distribution model of wading birds. Responses are calculated with all other model variables held at their mean. The black line represents the posterior mean and the gray shading represents the 95% posterior interval.

Generally, the spatial effects (easting and northing) were important parameters within the model for all species. Calculated Bayes factors indicated that the easting coordinate provided a particularly strong effect for the great blue heron, great egret, small dark herons, small white herons, roseate spoonbill, and white ibis (Table 3, values > 4.61). For all species except the little blue heron, moving eastward increased the probability of occupancy for the species (Fig 9). The effect of the northing coordinate was strong for each species with Bayes factors > 4.61 in all cases (Table 3). Again, most species exhibited a similar pattern of decreasing probability of occupancy as the northing coordinate increased (Fig 10). The little blue heron displayed the opposite trend.

**Fig 11:**
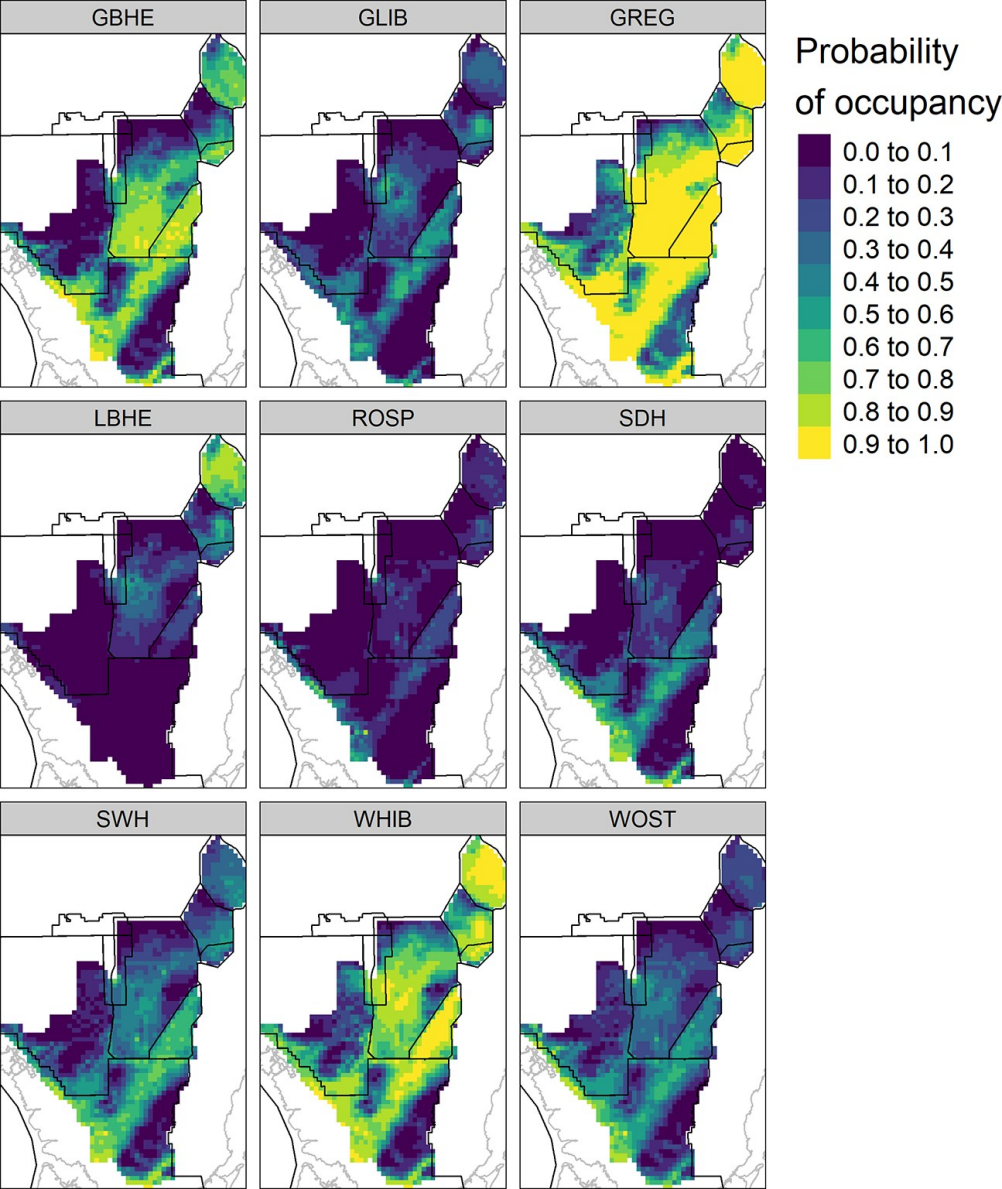
Example species-specific occupancy predictions. Species-specific predicted probabilities of occupancy across the Everglades landscape during 2009 calculated using the posterior mean of model parameters from a fitted joint species distribution model. Species are: GBHE (great blue heron); GLIB (glossy ibis); GREG (great egret); LBHE (little blue heron); ROSP (roseate spoonbill); SDH (small dark heron); SWH (small white heron); WHIB (white ibis); WOST (wood stork).

We generated species-specific maps of predicted occupancy for the year 2009 to show an example visualization that may be used by Everglades resource managers to plan restoration projects (Fig 11).

## Discussion

Our improved wading bird model revealed species-specific relationships between hydrology and wading bird presence on the landscape. Average depth during the breeding season was an important variable for all species. Great blue herons, great egrets, little blue herons, and small white herons had a positive relationship between water depths and probability of presence, a trend that is consistent with much of the literature [22, 23, 26]. Herons are visual feeders that strike their fish prey from above, making them generally tolerant of relatively deeper water. Conversely, the wood stork, small dark herons, and glossy ibis had a negative relationship with average water depth, with probability of occupancy decreasing at deeper water depths. This also coincides with the ecology of these species, as ibis and storks are filter feeders that use their bill to ‘feel’ prey within the water. Thus, ibis and storks are generally more successful foragers in shallow water [23, 26]. Tricolored herons, the species that dominates the small dark heron group, are known to be more tolerant of drought conditions than other larger bodied herons [47].

Average water recession rate over the breeding season was important for all species in the model and all species except the glossy ibis and little blue heron had a higher probability of occupancy when water receded between an average of 0.5 cm and 0 cm per day. Glossy ibis and little blue heron exhibited the opposite response to water recession rates, increasing occupancy after water trended toward increased between > 0 and 0.5 cm per day. Glossy ibis have been shown to be tolerant of moderately deep water compared to white ibis and wood stork [21], but also may prefer to feed on submerged vegetation in moderately deep waters as opposed to accessing crayfish in shallow waters [48]. The little blue heron relationship had much wider credible intervals than all other species. This could be a function of the little blue heron’s plasticity when it comes to water depths during foraging. Previous studies have shown little blue herons are not particularly constrained by deeper waters and this may be why they are more tolerant of increasing water depths during the breeding season [21].

All species exhibited a similar response to the number of dry days over the last 3 years, where probability of occupancy was highest at 0 dry days, dropping off precipitously around 300 dry days. Most vegetation in the Everglades is adapted to the dynamic pattern of drying and flooding that occurs, but longer periods of drought or flooding can change the vegetation present on the landscape over time [49]. Shorter hydroperiods can lead to the loss of slough habitat important for wading bird foraging [26] or facilitate the invasion of cattail which can cause rapid peat accumulation that makes sites more susceptible to drying and long-term vegetation changes [50]. Thus, it is not surprising that our models show high probabilities of occupancy of all wading birds at lower numbers of dry days over a 3-year period.

All species except for the glossy ibis displayed a similar response to proportion canopy cover. For most species, higher amounts of canopy cover increased the probability of occupancy. This is consistent with the ecology of wading birds generally; these species use woody vegetation for nesting and thus should be associated with areas that have greater canopy cover [27, 33]. However, it is important to note that proportion of canopy cover never reached above ~0.65 on the landscape. Wading birds do need open areas of marsh for feeding, therefore if this metric was assessed at a smaller scale, we likely would have seen wading bird occupancy peak at an optimal proportion of canopy cover and then decline at higher proportions up to 1. Glossy ibis showed a slight decline with increases in canopy cover, but this relationship was not strong and may not be ecologically meaningful.

The spatial parameters we included within the model, easting and northing coordinate, both exhibited strong influence on the probability of occupancy of all species. This was not surprising, as we included these predictors to account for residual spatial autocorrelation within the data due to covariates we could not measure and evidence from a previous study that indicated spatial autocorrelation is likely present in the data [7].

Detection probabilities of each wading bird species were generally low but ranged between 0.042 (roseate spoonbills and little blue herons) to 0.511 (great egrets). These detection probabilities are slightly lower than those assessed in a previous exploration of detection probability from a subset of data from the same SRF surveys, but general species-specific patterns are the same [20]. Conroy et al. [20] did suggest that their estimated detection probabilities were likely higher than the true detection probability of each species. Thus, we believe our model’s estimation of species-specific detection probabilities are reasonable even though we did not include covariates on species detection.

While the diagnostic plots of model fit for each species did not reveal fit issues, the AUC values for all species hovered around 0.7 except for the great egret, the most detected species within the dataset. While an AUC of 0.7 is still an adequately accurate model, there may be reasons why AUC values were not higher. First, the environmental covariates were averaged onto on a 2-by-2 km grid to match with the spatial grain of detection data. In the Everglades system, small areas of topography changes can create hydrologic patterns that would not be reflected in a 2 km resolution. Bird presence or absence could be due to conditions that we were unable to capture given the data available. It is also probable that we are missing an important driver of wading bird occupancy as an environmental covariate. For example, EDEN hydrologic output does not extend to coastal tidal and estuarine areas which are likely important foraging grounds for wading birds during the breeding season. While the hydrologic drivers included within the model explain prey availability to wading bird foraging, we do not have landscape-scale information on potential prey densities across the landscape. There are some models of fish density response to hydrologic drivers within the Everglades landscape, but these models are site-specific and cannot be scaled up accurately to the resolution we have modeled here [51]. Similarly, crayfish are a significant part of many wading bird species diets in the Everglades [52], but no landscape-scale information on their populations or response to hydrology at the landscape-scale is yet available to relate to wading bird presence or breeding success [53]. An important next step for improving models of wading bird occurrence in the Everglades would be to develop dynamic information on fish and crayfish densities across the system at larger scales. Similarly, wading birds may be responding to the presence of other species within the system, such as alligators. Alligators may serve as ‘nest-protectors’ from meso-carnivores and wading birds could be selecting for areas where alligators are present to capitalize on this protection during the nesting season [54].

Though it is possible to estimate residual correlation and infer species interactions using joint species distribution models [19], we opted not to estimate these for several reasons. Most importantly, including residual correlation estimation resulted in models that would not converge. If we were able to examine residual correlation between species, it is likely we would have observed moderate to high correlation among all wading bird species based on the overall similarities that species showed to the modeled environmental covariates. These similarities are expected, as wading birds in the Everglades ecosystem often forage in mixed flocks [5]. Finally, it is important to note that recently there has been some debate on whether examining co-occurrence of species can be used as evidence of ecological interactions [55]. To make proper conclusions about species interactions, it is important that the sampling resolution can capture both negative and positive interactions [56] and that the entire distributional range of each species is sampled [55]. The distributions of all species modeled here extend outside the spatial extent of the Everglades, therefore conclusions about species interactions using this model could be erroneous.

Restoration implementation and seasonal water management decisions in the Everglades rely heavily on models of indicator species responses to simulated changes in hydrology within the system. We used new developments in species distribution modeling methodology to improve our current models of wading bird distributions in three ways. First, we used a detection-nondetection modeling framework that produced output on a 0–1 scale of probability of occupancy. The output thus becomes intuitive for decision makers and comparable across species as there is a defined lower and upper limit that is consistent across species. Second, we expanded the number of wading bird species decision makers or managers could consider from three to nine by using a joint species framework. Through use of community-level priors in the Bayesian analysis, we could model rarer species such as the roseate spoonbill or glossy ibis, species typically not detected enough for meaningful modeling to occur. Considering the response of both common and rare wading bird species to changes in hydrology gives decision makers a broader view of the wading bird community. This also resulted in the first statistical landscape-scale models of the small white heron and small dark heron for use in restoration decision making (but see [57] for a rules-based approach). Third, our model accounts for species-specific detectability. Accounting for species detection probability in models of species distribution is vitally important, as not doing so can bias predicted occupancy estimates and provide misleading information to decision makers [8–12]. Using the relationships between hydrology and species occupancy measured from the fitted model, we can generate predicted occupancy maps for each species and compare across simulated hydrologic scenarios for restoration planning [58, 59]. This process typically involves assessing predicted species occupancy for a baseline and alternative hydrologic scenario, then comparing the percent differences in predicted occupancy from the baseline to the alternative. Decision-makers can then use the relative differences across the landscape and through time as quantitative metrics of improvements and declines in predicted occupancy of each species. Depending on the values of decision-makers, one or more alternatives will be preferred, and these preferences can feed into further refinement and analysis of scenario performance in a structured decision making context [60].

## Supporting information

S1 Appendix JAGS code used to construct a multi-species occupancy model of Everglades wading birds. (DOCX)

## References

[1] Lodge TE. The Everglades Handbook: Understanding the Ecosystem. 4th ed Boca Raton: CRC Press 2016.

[2] Sklar FH, Chimney MJ, Newman S, McCormick P, Gawlik D, Miao S, et al The ecological-societal underpinnings of Everglades restoration. Front Eco Env. 2005; 3: 161–169.

[3] LoSchiavo AJ, Best RG, Burns E, Gray S, Harwell MC, Hines B, et al Lessons learned from the first decade of adaptive management in comprehensive Everglades restoration. Eco Soc. 2013; 18: 70.

[4] Frederick PC, Gawlik DE, Ogden JC, Cook MI, and Lusk M. The white ibis and wood stork as indicators for restoration of the Everglades ecosystem. Eco Ind. 2009; 9: S83–S95.

[5] Ogden JC. A comparison of wading bird nesting colony dynamics (1931–1946 and 1974–1989) as an indication of ecosystem conditions in the southern Everglades In: Davis SM and Ogden JC, editors. Everglades, the ecosystem, and its restoration. CRC Press; 1994 pp. 533–570.

[6] Crozier GE and Gawlik DE. Wading bird nesting effort as an index to wetland system integrity. Waterbirds. 2003; 26:303–324.

[7] Beerens JM, Noonburg EG, and Gawlik DE. Linking dynamic habitat selection with wading bird foraging distributions across resource gradients. PLoS ONE. 2015; 10: e0128182 10.1371/journal.pone.0128182 26107386 PMC4480858

[8] MacKenzie DI, Nichols JD, Lachman GB, Droege S, Royle JA, and Langtimm CA. Estimating site occupancy rates when detection probabilities are less than one. Ecology. 2002; 83:2248–2255.

[9] Gu W and Swihart RH. Absent or undetected? Effects of non-detection of species occurrence on wildlife-habitat models. Bio Cons. 2004; 116:195–203.

[10] Ruiz-Gutiérrez V and Zipkin EF. Detection biases yield misleading patterns of species persistence and colonization in fragmented landscapes. Ecosphere. 2011; 2:1–14.

[11] Lahoz-Monfort JJ, Guillera-Arroita G and Wintle BA. Imperfect detection impacts the performance of species distribution models. Glob Eco Biogeo. 2013; 23:504–515.

[12] Guillera-Arroita G. Modelling of species distributions, range dynamics and communities under imperfect detection: advances, challenges, and opportunities. Ecography. 2016; 40:281–295.

[13] Gaston KJ and Fuller RA. Commonness, population depletion and conservation biology. Trends Eco Evo. 2008; 23:14–19. 10.1016/j.tree.2007.11.001 18037531

[14] Godsoe W and Harmon LJ. How do species interactions affect species distribution models? Ecography. 2012; 35:811–820.

[15] Zipkin EF, Royle JA, Dawson DK, and Bates S. Multi-species occurrence models to evaluate the effects of conservation and management. Bio Cons. 2010; 143:479–484.

[16] Rota CT, Ferreira MAR, Kays RW, Forrester TD, Kalies EL, McShea WJ, et al A multispecies occupancy model for two or more interacting species. Meth Eco Evo. 2016; 7:1164–1173.

[17] Pollock LJ, Tingley R, Morris WK, Golding N, O’Hara RB, Parris KM, et al Understanding co-occurrence by modelling species simultaneously with a Joint Species Distribution Model (JSDM). Meth Eco Evo. 2014; 5:397–406.

[18] Warton DI, Guillaume-Blanchet F, O’Hara RB, Ovaskainen O, Taskinen S, Walker SC, et al So many variables: joint modeling in community ecology. Trends Eco Evo. 2015; 30:766–779. 10.1016/j.tree.2015.09.007 26519235

[19] Tobler MW, Kéry M, Hui FKC, Guillera-Arroita G, Knaus P, and Sattler T. Joint species distribution models with species correlations and imperfect detection. Ecology. 2019; 100:e02754 10.1002/ecy.2754 31062356

[20] Conroy MJ, Peterson JT, Bass OL, Fonnesbeck CJ, Howell JE, Moore CT, et al Sources of variation in detection of wading birds from aerial surveys in the Florida Everglades. The Auk. 2008; 125:731–743.

[21] Gawlik DE. The effects of prey availability on the numerical response of wading birds. Eco Mono. 2002; 72:329–346.

[22] Lantz SM, Gawlik DE, and Cook MI. The effects of water depth and submerged aquatic vegetation on the selection of foraging habitat and foraging success of wading birds. The Condor. 2010; 112:460–469.

[23] Beerens JM, Gawlik DE, Herring G, and Cook MI. Dynamic habitat selection by two wading bird species with divergent foraging strategies in a seasonally fluctuating wetland. The Auk. 2011; 128:651–662.

[24] Herring G, Gawlik DE, Cook MI, and Beerens JM. Sensitivity of nesting great egrets (Ardea alba) and white ibises (Eudocimus albus) to reduced prey availability. The Auk. 2010; 127:660–670.

[25] Telis PA, Xie Z, Liu Z, Li Y, and Conrads PA. The Everglades Depth Estimation Network (EDEN) surface-water model, version 2: U.S. Geological Survey Scientific Investigations Report 2014–5209.

[26] Bancroft GT, Gawlik DE, and Rutchey K. Distribution of wading birds relative to vegetation and water depths in the northern Everglades of Florida, USA. Waterbirds. 2002; 25:265–277.

[27] Pierce RL and Gawlik DE. Wading bird foraging habitat selection in the Florida Everglades. Waterbirds. 2010; 33:494–503.

[28] Botson BA, Gawlik DE, and Trexler JC. Mechanisms that generate resource pulses in a fluctuating wetland. PLoS ONE. 2016; 11:e0158864 10.1371/journal.pone.0158864 27448023 PMC4957811

[29] Ross MS, Reed DL, Sah JP, Ruiz PL, and Lewin MT. Vegetation:environment relationships and water management in Shark Slough, Everglades National Park. Wet Eco Manag. 2003; 11:291–303.

[30] Todd MJ, Muneepeerakul R, Pumo D, Azaele S, Miralles-Wilhelm F, Rinaldo A, et al Hydrological drivers of wetland vegetation community distribution within Everglades National Park, Florida. Adv Water Res. 2010; 33:1279–1289.

[31] Cook MI, Call EM, Kobza RM, Hill SD, and Saunders CJ. Seasonal movements of crayfish in a fluctuating wetland: implications for restoring wading bird populations. Fresh Bio. 2014; 59:1608–1621.

[32] USDA Forest Service. NLCD 2011 Tree Canopy Cover (CONUS). 2019. Salt Lake City, UT.

[33] Chastant JE, Peterson ML, and Gawlik DE. Nesting substrate and water-level fluctuations influence wading bird nesting patterns in a large shallow eutrophic lake. Hydrobiologica. 2017; 788:371–383.

[34] Devarajan K, Morelli TL, and Tenan S. Multi-species occupancy models: review, roadmap, and recommendations. Ecography. 2020; 10.1111/ecog.04902 33304029 PMC7116457

[35] Dorazio RM and Royle JA. Estimating size and composition of biological communities by modeling the occurrence of species. J Amer Stat Ass. 2005; 100:389–398.

[36] R Core Team. R: A Language and Environment for Statistical Computing. R Foundation for Statistical Computing Vienna; 2019 https://www.R-project.org.

[37] Plummer M. JAGS: A program for analysis of Bayesian graphical models using Gibbs sampling. Proc 3^rd^ Int Work Dist Stat Comp; 2003 https://mcmc-jags.sourceforge.net.

[38] Su Y and Yajima M. R2jags: Using R to Run ‘JAGS’. R package version 0.5–7; 2015 https://CRAN.R-project.org/package=R2jags.

[39] Gelman A, Carlin JB, Stern HS, and Rubin DB. Bayesian data analysis. Florida: CRC Press; 2014.

[40] Dunn PK, and Smyth GK. Randomized quantile residuals. J Comp Graph Stat. 1996; 5:236–244.

[41] Warton DI, Stoklosa J, Guillera-Arroita G, MacKenzie DI, and Welsh AH. Graphical diagnostics for occupancy models with imperfect detection. Meth Eco Evo. 2017; 8:408–419.

[42] Jiménez-Valverde A. Insights into the area under the receiver operating characteristic curve (AUC) as a discrimination measure in species distribution modelling. Glob Eco Biog. 2012; 21:498–507.

[43] Zipkin EF, Campbell Grant EH, and Fagan WF. Evaluating the predictive abilities of community occupancy models using AUC while accounting for imperfect detection. Eco App. 2012; 22:1962–1972. 10.1890/11-1936.1 23210312

[44] Jeffreys H. Theory of probability. 1961; Oxford, UK: Oxford University Press.

[45] Verdinelli I and Wasserman L. Computing bayes factors using a generalization of the Savage-Dickey density ratio. J Amer. Stat. Ass. 1995; 90:614–618.

[46] Makowski D, Ben-Shachar MS, and Lüdecke D. bayestestR: Describing effects and their uncertainty, existence and significance within the Bayesian framework. J. Open Source Soft. 2019; 4:1541.

[47] Strong AM, Bancroft GT, and Jewell SD. Hydrological constraints on tricolored heron and snowy egret resource use. The Condor. 1997; 99:894–905.

[48] Acosta M, Mugica L, Mancina C, and Ruiz X. Resource partitioning between Glossy and White ibises in a rice field system in southcentral Cuba. Col Waterbirds. 1996; 19:65–72.

[49] Sah JP, Ruiz PL, and Ross MS. Spatio-temporal pattern of plant communities along a hydrologic gradient in Everglades tree islands. For Eco Manage. 2018; 421:16–31.

[50] Bansal S, Lishawa SC, Newman S, Tangen BA, Wilcox D, Albert D, et al Typha (cattail) invasion in North American wetlands: biology, regional problems, impacts, ecosystem services, and management. Wetlands. 2019; 39:645–684.

[51] Trexler JC and Goss CW. Aquatic fauna as indicators for Everglades restoration: applying dynamic targets in assessments. Eco Ind. 2009: 9: S108–S119.

[52] Boyle RA, Dorn NJ, and Cook MI. Nestling diet of three sympatrically nesting wading bird species in the Florida Everglades. Waterbirds. 2012; 35:154–159.

[53] Frederick PC and Spalding MG. Factors affecting reproductive success by wading birds (Ciconiiformes) in the Everglades ecosystem In: Davis SM and Odgen JC, editors. The Everglades: the ecosystem and its restoration. Delray Beach: CRC Press; 1994 pp 659–691.

[54] Burtner BF and Frederick PC. Attraction of nesting wading birds to alligators (Alligator mississippiensis). Testing the ‘nest protector’ hypothesis. Wetlands. 2017; 37:697–704.

[55] Blanchet FG., Cazelles K, and Gravel D. Co-occurrence is not evidence of ecological interactions. Ecology Letters. 2020; 23:1050–1063. 10.1111/ele.13525 32429003

[56] Araújo MB, and Rozenfeld A. The geographic scaling of biotic interactions. Ecography. 2014; 37:001–010.

[57] DeAngelis DL, Gross LJ, Huston MA, Wolff WF, Fleming DM, Comiskey EJ, et al Landscape modeling for Everglades Restoration. Ecosystems. 1998; 1:64–75.

[58] Catano CP, Romañach SS, Beerens JM, Pearlstine LG, Brandt LA, Hart KM, et al Using scenario planning to evaluate the impacts of climate change on wildlife populations and communities in the Florida Everglades. Env Manage. 2014; 55:807–823. 10.1007/s00267-014-0397-5 25371194

[59] Pearlstine LG, Beerens JM, Reynolds G, Haider SM, McKelvy M, Suir K, et al Near-term spatial hydrologic forecasting in Everglades, USA for landscape planning and ecological forecasting. Envir. Model. & Soft. 2020; 104783.

[60] National Academies of Sciences, Engineering, and Medicine. Progress Toward Restoring the Everglades: The Seventh Biennial Review-2018. National Academies Press; 2019.

