# Supplementary material for: Joint species distribution models of Everglades wading birds to inform restoration planning
Source: PLoS One. 2021 Jan 28;16(1):e0245973. doi: 10.1371/journal.pone.0245973 (PMC7842948; doi:10.1371/journal.pone.0245973)
Supplement: S1 Appendix — (DOCX) [file pone.0245973.s001.docx]

**S1 Appendix. JAGS code used to construct a multi-species occupancy model of Everglades wading birds.**

model{

#Priors

#Hyperpriors - community-level prior distributions

# occupancy community level priors

a0.mean ~ dunif(0,1)

mu.a0 <- log(a0.mean) - log(1-a0.mean)

tau.a0 ~ dgamma(0.1,0.1)

#detection level community priors

b0.mean ~ dunif(0,1)

mu.b0 <- log(b0.mean) - log(1-b0.mean)

tau.b0 ~ dgamma(0.1,0.1)

#covariate community priors

mu.a1 ~ dnorm(0,0.001) #depth_breed

mu.a2 ~ dnorm(0,0.001) #depth_breed^2

mu.a3 ~ dnorm(0,0.001) #rec

mu.a4 ~ dnorm(0,0.001) #rec^2

mu.a5 ~ dnorm(0,0.001) #daysdry

mu.a6 ~ dnorm(0,0.001) #daysdry^2

mu.a7 ~ dnorm(0,0.001) #canopy

mu.a8 ~ dnorm(0,0.001) #utm_x

mu.a9 ~ dnorm(0,0.001) #utm_y

tau.a1 ~ dgamma(0.1,0.1)

tau.a2 ~ dgamma(0.1,0.1)

tau.a3 ~ dgamma(0.1,0.1)

tau.a4 ~ dgamma(0.1,0.1)

tau.a5 ~ dgamma(0.1,0.1)

tau.a6 ~ dgamma(0.1,0.1)

tau.a7 ~ dgamma(0.1,0.1)

tau.a8 ~ dgamma(0.1,0.1)

tau.a9 ~ dgamma(0.1,0.1)

# species-specific priors from community priors

for (i in 1:nspec){

a0[i] ~ dnorm(mu.a0, tau.a0)

b0[i] ~ dnorm(mu.b0, tau.b0)

a1[i] ~ dnorm(mu.a1, tau.a1)

a2[i] ~ dnorm(mu.a2, tau.a2)

a3[i] ~ dnorm(mu.a3, tau.a3)

a4[i] ~ dnorm(mu.a4, tau.a4)

a5[i] ~ dnorm(mu.a5, tau.a5)

a6[i] ~ dnorm(mu.a6, tau.a6)

a7[i] ~ dnorm(mu.a7, tau.a7)

a8[i] ~ dnorm(mu.a8, tau.a8)

a9[i] ~ dnorm(mu.a9, tau.a9)

# estimate true occurrence for species i at site j

for (j in 1:nsite) {

logit(psi[j,i]) <- a0[i] + a1[i] * depth_breed[j] +

a2[i] * depth_breed_sq[j] +

a3[i] * recession[j] +

a4[i] * recession_sq[j] +

a5[i] * daysdry[j] +

a6[i] * daysdry_sq[j] +

a7[i] * canopy[j] +

a8[i] * utm_x[j] +

a9[i] * utm_y[j]

mu.psi[j,i] <- psi[j,i]

Z[j,i] ~ dbern(mu.psi[j,i])

#estimate detection for species i at site j during survey k

for (k in 1:nrep){

logit(p[j,k,i]) <- b0[i]

mu.p[j,k,i] <- p[j,k,i] * Z[j,i]

X[j,k,i] ~ dbern(mu.p[j,k,i])

} #k

} #j

} #i

}
